# The diversity of opsins in Lake Baikal amphipods (Amphipoda: Gammaridae)

**DOI:** 10.1186/s12862-021-01806-9

**Published:** 2021-05-10

**Authors:** Polina Drozdova, Alena Kizenko, Alexandra Saranchina, Anton Gurkov, Maria Firulyova, Ekaterina Govorukhina, Maxim Timofeyev

**Affiliations:** 1 Irkutsk State University, Irkutsk, Russia; 2 Baikal Research Centre, Irkutsk, Russia; 3 Bioinfomatics Institute, St. Petersburg, Russia; 4 Computer Technologies Department, ITMO University, St. Petersburg, Russia

**Keywords:** Crustacea: Malacostraca: Amphipoda, Lake Baikal, Ancient ecosystems, Vision, Parallel evolution

## Abstract

**Background:**

Vision is a crucial sense for the evolutionary success of many animal groups. Here we explore the diversity of visual pigments (opsins) in the transcriptomes of amphipods (Crustacea: Amphipoda) and conclude that it is restricted to middle (MWS) and long wavelength-sensitive (LWS) opsins in the overwhelming majority of examined species.

**Results:**

We evidenced (i) parallel loss of MWS opsin expression in multiple species (including two independently evolved lineages from the deep and ancient Lake Baikal) and (ii) LWS opsin amplification (up to five transcripts) in both Baikal lineages. The number of LWS opsins negatively correlated with habitat depth in Baikal amphipods. Some LWS opsins in Baikal amphipods contained MWS-like substitutions, suggesting that they might have undergone spectral tuning.

**Conclusions:**

This repeating two-step evolutionary scenario suggests common triggers, possibly the lack of light during the periods when Baikal was permanently covered with thick ice and its subsequent melting. Overall, this observation demonstrates the possibility of revealing climate history by following the evolutionary changes in protein families.

**Supplementary Information:**

The online version contains supplementary material available at 10.1186/s12862-021-01806-9.

## Background

Vision has been a crucial sense for the evolutionary success of many animal groups. Yet, the majority of animal vision systems are quite similar at the molecular level, as they comprise opsin proteins covalently bound to a chromophore molecule (aldehyde derivatives of retinol) [1, 2].

Opsins form a monophyletic group within the G-coupled receptor superfamily and possess a characteristic conserved lysine residue in the seventh transmembrane domain (Lys^7.43^, or Lys^296^ in the bovine rhodopsin), to which retinal is attached via a protonated Schiff base [3]. They are further subdivided into four major groups (major eumetazoan opsin paralogs), one of which includes canonical visual ciliary (c) opsins and rhabdomeric (r) opsins [4]. At least four groups of animals (spiders; Pancrustacea, i.e. insects and crustaceans; cephalopods; and chordates) independently developed complex visual systems. Many insects and crustaceans possess compound eyes equipped with r-opsins [2, 4, 5].

Insect opsins include at least five classes, at least three of which are directly connected to vision: green-sensitive (or long wavelength-sensitive, LWS), blue-sensitive (also short wavelength-sensitive, SWS), and UV-sensitive (UVS) opsins [6–9]. LWS opsins are most frequently duplicated, while SWS opsins are most frequently lost. Sometimes duplicated LWS or UVS genes accumulate mutations that may allow their products to compensate for the loss of SWS opsins [4, 8].

The diversity of opsins in the set of taxa traditionally referred to as Crustacea is also very wide and probably incompletely understood, similar to their phylogenetic relationships. Most recent phylogeny-based classifications of visual r-opsins in crustaceans includes SWS/UVS, which are considered a single class, middle wavelength-sensitive (MWS), and LWS opsins [10–12], even though sometimes authors consider MWS and SWS/UV the same clade, naming it SWS [13–15]. The approximate minimal spectral sensitivity of LWS pigments is around 490 nm [13, 16]; there is yet not enough spectral data to delimit MWS and LWS opsins. Interestingly, one of the first opsin sequencing studies found two MWS opsins in the eyes of a brachyuran crab *Hemigrapsus sanguineus* with a resulting spectral maximum of the combined eye around 480 nm [17]. Overall, there is still not enough accumulated evidence to bridge the gap between the experimental spectral characteristics of crustacean photoreceptors and opsin sequences.

The most speciose taxa within Crustacea include Branchiopoda (the best-known genera are *Daphnia*, *Triops*, and *Artemia*), Copepoda (*e.g.*, the genera *Tigriopus* and *Calanus*), Thecostraca (includes barnacles), and the largest group, Malacostraca [18, 19]. The genomes of model brachiopod species, *D. pulex* and *D. magna*, encode 48 and 32 opsins, respectively, including LWS (25 and 12, respectively), blue-sensitive, UV-sensitive, and other classes (including non-visual) [20]. In other brachiopods species (*Triops granarius*, *T. longicaudatus*, and *Brachinella kugenumaensis*), multiple SWS/UV opsin transcripts were found with targeted amplification with degenerate primers [21]. Copepods express mostly visual MWS opsins and C-type pteropsins (most likely non-visual) [22].

**Fig. 1:**
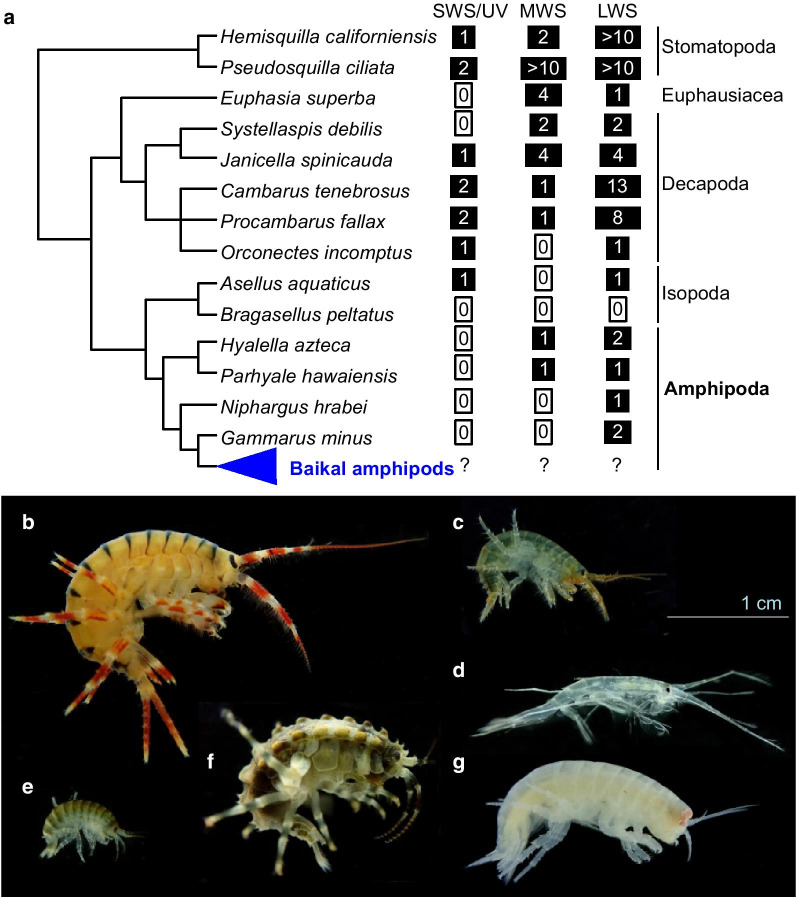
The diversity of visual opsins in selected representatives of Eumalacostraca and examples of Lake Baikal amphipods. **a** Visual opsin diversity in selected malacostracan species: *Hemisquilla californiensis* and *Pseudosquilla ciliata* (Stomatopoda) [10]; *Euphausia superba* (Euphausiaceae) [23]; *Janicella spinicauda*, *Systellaspis debilis*, *Cambarus tenebrosus*, *Procambarus fallax*, and *Orconectes incomptus* (Decapoda) [11, 12, 24]; *Hyalella azteca*, *Parhyale hawaiensis*, *Niphargus hrabei*, and *Gammarus minus* (Amphipoda) [14, 16, 25, 26]. In the case of *Janicella spinicauda*, both photophore and eye opsins were counted. **b**–**g** Examples of Lake Baikal amphipods and their ecological characteristics [27]. **b** *Eulimnogammarus maackii* (Gersfteldt, 1858), a benthic species mostly found at depths of 0–40 m. **c** Another benthic species, *E. cyaneus* (Dybowsky, 1874), mostly concentrating close to the shoreline. **d** The only pelagic species *Macrohectopus branickii* (Dybowsky, 1874). **e** Another littoral benthic species *Gmelinoides fasciatus* (Stebbing, 1899), a unique species which originated in Baikal but was successfully introduced into multiple water bodies in Siberia and European Russia [28]. **f** *Brandtia latissima latior* (Dybowsky, 1874) mostly found at depths from 0.5 to 50 m. **g** A deep-water eurybathic scavenger *Ommatogammarus albinus* (Dybowsky, 1874) mainly found below 200 m. Note the presence of large pigmented eyes in the deep-water species

The Malacostraca include Leptostraca, Stomatopoda, Anaspidaceae, Euphausiacea, Decapoda, Mysida, Tanaidacea, Isopoda, Cumacea, and Amphipoda, with the latter four united to Peracaridae [19]. An overview of visual opsin diversity in malacostracan transcriptomes is presented in Fig. 1A.

Many mantis shrimps (Stomatopoda) species express multiple SWS, MWS, and LWS opsins in their retinas [10, 29], even though monochromatic species with only LWS opsins are also known [30]. Studying expression patterns of the exceptionally diverse opsin transcripts in the stomatopod species *Neogonodactylus oerstedii* has revealed complex spatial patterns and is a promising approach towards understanding the functions of multiple opsins within one eye [29]. The model Euphausiacea species, *Euphausia superba* (Antarctic krill), expresses at least one LWS opsin and multiple MWS opsin transcripts [23]. The sequence diversity of opsins in Mysida has only been explored with targeted amplification using genomic DNA as a template, and multiple LWS opsins were found [31, 32]. Within Decapoda, the diversity of opsins has been explored in the eyes and bioluminescent organ of Oplophoridae shrimps and one non-bioluminescent shrimp belonging to the Benthesicymidae family [11–13]; in the genome of a Penaeidae shrimp *Litopenaeus vannamei*, in which the opsin family is significantly expanded [33]; and in the eyes of 14 cave and surface crayfish species of the Cambaridae family [24]; LWS opsins were found in all of the studies species, while some species lacked either MWS or SWS opsins. The opsins of Isopoda have been mostly explored in the context of cave adaptation [14, 34]; the diversity of opsins ranged from complete absence (in a surface and two subterranean species) to one SWS/UV and one LWS opsin (surprisingly conserved in the cave populations in this species).

The diversity of opsins in the large group of amphipods (almost 10,000 species known [35]) is much less known and probably not so wide. Transcriptomes or genomes of only four species have been specifically explored. The exploration of the *Hyalella azteca* genome revealed two LWS opsins and the first amphipod MWS opsin characterized [25]; a subsequent transcriptome exploration revealed three LWS opsins and one MWS opsin [15]. A comprehensive study of the visual system in *Parhyale hawaiensis* revealed one LWS and one MWS opsin [16]. Studies of cave populations of *Gammarus minus* and *Niphargus hrabei* only revealed the presence of LWS opsins (two genes and three contigs, respectively) [14, 26, 36]. In the case of *G. minus*, both cave and surface populations were studied, and their representatives had identical diversity of opsins but dramatic differences in their expression levels [26]. However, each of these studies analyzed a particular species (either emerging model objects or species with cave populations). At the same time, amphipods include closely related species flocks such as Ponto-Caspian and Baikal groups [37].

Lake Baikal amphipods are of particular interest due to their morphological diversity, close relatedness, and heterogeneous habitats (Fig. 1b–g). Moreover, Lake Baikal is the only freshwater habitat with deep-water fauna [38, 39]. The lake is home for several endemic species flocks, including one vertebrate group (sculpins, or cottoid fishes), several crustacean lineages (amphipods, the largest species group, as well as ostracods and isopods), gastropods, oligochaetes, and flatworms [40, 41]. Studies of the sculpin visual pigments revealed that their absorbance maxima shift towards shorter wavelengths with increasing habitat depth [42] and identified key amino substitutions responsible for spectral tuning [43, 44]. Interestingly, the most probable reason for the selection of these shifts is not matching the downwelling light but filtering of photoreceptor noise to increase performance at extremely low-light conditions [43].

However, visual systems of Baikal amphipods, which also inhabit all depths of the lake and co-evolved with their main predators, sculpins [40], have not been explored yet. It is well-known that these amphipods respond to light with diel vertical migrations [45, 46], but no molecular-level information of opsin sequences has been published so far. Importantly, valuable transcriptomic resources for amphipod species from Lake Baikal have recently become available [47, 48]. By integrating these and other data sources, we explore the diversity of opsins in Baikal and other amphipods in connection to their habitats and evolutionary histories.

## Results

### The diversity of opsin transcripts in different phylogenetic lineages of amphipods suggests multiple losses of MWS opsins

To obtain the first idea of visual opsin diversity in the transcriptomes of endemic Baikal amphipods, we searched for opsin transcripts in the 64 published transcriptome assemblies [47] with PIA3, a pipeline we modified from PIA2 [15]. The modifications allowed us to automatically retrieve opsin class information and optionally discard short sequences, as those can be prone to misclassification. This analysis only revealed LWS opsins (from 1 to 4 unique transcripts in different species); in 21 of 64 assemblies, no opsin transcripts were found (Additional file 1: Table S1).

Apart from true lack of MWS opsin expression, the result may have been caused by (1) the lack of eye material in the sample (as for animals larger than 3 mm mesosome cross-sections were used [47]), (2) contig filtering, or (3) particularly poor assembly of other opsins (for example, caused by low expression of other classes of opsins).

The first hypothesis (absence of eye tissues in the RNA sequencing material as the reason for the lack of MWS opsins) was tested by including in the analysis *E. verrucosus*, *E. cyaneus*, and *G. lacustris* assemblies based on whole-body material [48], which also returned only LWS opsins (Additional file 1: Table S1).

The second hypothesis on the possible effect of contig filtering opportunity was tested by reassembling the transcriptomes with Trinity and rnaSPAdes and running PIA3 on new unfiltered assemblies. The second assembler was added due to the fact that it produces fewer similar isoforms [49] and also was found to work very fast on these relatively low-coverage data. We found that rnaSPAdes produced generally better assemblies in terms of recovered arthropod conservative genes and the number of one-copy BUSCOs (Fig. S1A-E in Additional file 2: Figs. S1–S7); the latter might be especially useful for full transcriptome-based phylogenetic reconstruction.

The number of found diverse opsin transcripts also increased in the reassembled transcriptomes (Fig. S1F in Additional file 2: Figs. S1–S7), but all of them still belonged to LWS opsins (Additional file 1: Table S1). As neither of the two reassembly methods offered a significant advantage in the number of found opsins but in some cases, they differed in the found opsins (Additional file 2: Fig. S1F), we decided to merge the sequences obtained from different assemblies of the same species (sequences with $>95\%$ identity were treated as one) to obtain the best possible estimate of the number of opsin transcripts.

To test the third hypothesis (low expression of MWS opsins in Baikal amphipods leading to the poor assembly of these transcripts), we needed to obtain the closest possible reference for MWS opsins. Thus, we checked available transcriptome assemblies of other representatives of the Gammaridae family. They included European freshwater gammarids *Echinogammarus berilloni* [50], *Ech. veneris* [51], *G. fossarum*, *G. pulex*, and *G. wautieri*, as well as *Marinogammarus marinus* [50], *G. chevreuxi* [52, 53]. To put the results into a wider perspective, we checked other available amphipod transcriptome assemblies: *Hirondellea gigas* [54], *Grandidierella japonica* [55], *Melita plumulosa* [56], *Talitrus saltator* (brain transcriptome) [57], *Hyalella azteca* [58, 59], and *Caprella* sp. [19]. The data for *G. minus* [36] and *Parhyale hawaiensis* (head transcriptome) [60], in which opsin diversity has been explored already, were added to the analysis to verify the method. Finally, some transcriptomes were reassembled if only raw data were provided by the authors, namely *G. pisinnus* [61], *Trinorchestia longiramus* [62], and *Gondogeneia antarctica* [63]. In each case, the assemblies were searched for visual opsin sequences with PIA3.

The obtained 146 sequences (Additional file 3: Text S1) were used to build a phylogenetic tree. The longest branches were additionally analysed by finding the most similar sequences in the NCBI nr database by using web BLAST interface (Additional file 2: Fig. S2A) and making pairwise alignment with bovine rhodopsin. Indeed, these sequences most probably represented an octopamine receptor and an adrenergic receptor, respectively, according the the BLAST analysis and did not contain a lysine residue in the position corresponding to Lys^296^ in bovine rhodopsin; thus, they were erroneously considered opsins by PIA3. However, we consider the overall specificity of PIA3 with the chosen parameters (2 false positives for 146 sequences) acceptable. MWS transcripts (always one transcript or one cluster of $>95\%$ identical sequences per species) were found in *Gon. antarctica*, all European Gammaridae species, and all Talitridae species but were absent from all explored Gammaridae species from Asia, including *G. pisinnus* and Palearctic *G. lacustris*. Besides, in the *T. saltator* assembly we found two sequences that most probably belonged to SWS/UV and vertebrate-like opsins, respectively. It might be connected to the fact that in this case a brain transcriptome (instead of whole-body) was sequenced with high coverage [57]. In most other non-Gammaridae species, only LWS opsins could be found (Additional file 1: Table S1).

To track the history of opsin loss and duplication, we reconstructed species phylogeny based on 420 predicted single-copy orthologs. For that, we chose 36 good-quality assemblies (over 50% complete BUSCOs) for one species per genus (excluding the formal genus *Gammarus*) to avoid artefacts due to biased taxonomic sampling. We used only rnaSPAdes assemblies to avoid the influence of the assembly method. In brief, we predicted proteins in transcriptome assemblies, grouped putative orthologs, selected one-to-one orthologs in all species, aligned each ortholog group separately, filtered and concatenated the alignments, and reconstructed the maximum likelihood phylogeny. The trimmed nucleotide alignment contained 455,402 sites (178,150 of them were parsimony informative), while the corresponding amino acid alignment contained 151,293 sites (38,042 of them were parsimony informative). The nucleotide-based and amino acid-based trees were constructed under the GTR+F+R4 and JTT+F+R5 models, respectively. For more details of the algorithms and parameters see the Materials and Methods section.

**Fig. 2:**
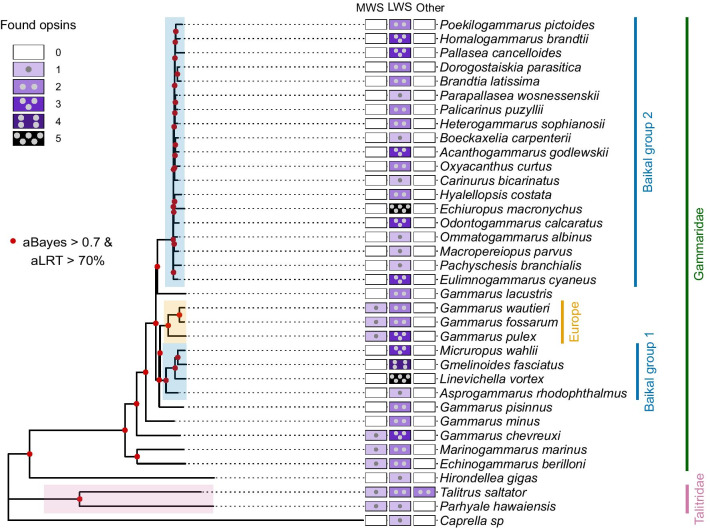
Multiple losses of MWS opsin expression in Baikal and other amphipods. Species phylogeny (based on one-copy orthologs present in all transcriptomes) is annotated with the number of opsins found. aLRT, approximate likelihood ratio test. aBayes, approximate Bayes test. Talitridae and Gammaridae are marked according to the World Amphipoda Database [66] accessed through the WoRMS database [67]

The topologies of the nucleotide-based (Fig. 2) and amino acid-based (Additional file 2: Fig. S2B) trees were identical and placed *G. lacustris* as a sister group to the second (younger) Baikal group, while European Gammarus species (*G. pulex*, *G. fossarum*, and *G. wautieri*) formed a sister clade to the first Baikal (more ancient) group. Baikal amphipods are known to include at least two phylogenetic lineages [47, 64] and have been recently suggested to have separated from the Palearctic *Gammarus* species early in their evolution [65].

Generally, our data did not contradict the phylogenetic reconstructions obtained earlier with selected markers [68–70] or whole transcriptome data [47] and deepen our understanding of the amphipod phylogeny. Taken together, the phylogeny and opsin diversity suggest that the last common amphipod ancestor most probably possessed one MWS opsin, and the loss of MWS opsins occurred multiple times. The presence of one MWS opsin at the root of the amphipod tree is also supported by the results of the ancestral state reconstruction analysis (Additional file 2: Fig. S2C). The most probable number of LWS opsins at the root of the amphipod tree could not be defined with the ancestral state reconstruction analysis, but the most probable number of LWS opsins in the last common gammarid ancestor equaled two (Additional file 2: Fig. S2D).

### Transcript abundance confirms the absence of MWS opsins in Baikal amphipods and reveals a difference in LWS and MWS opsin expression levels

**Fig. 3:**
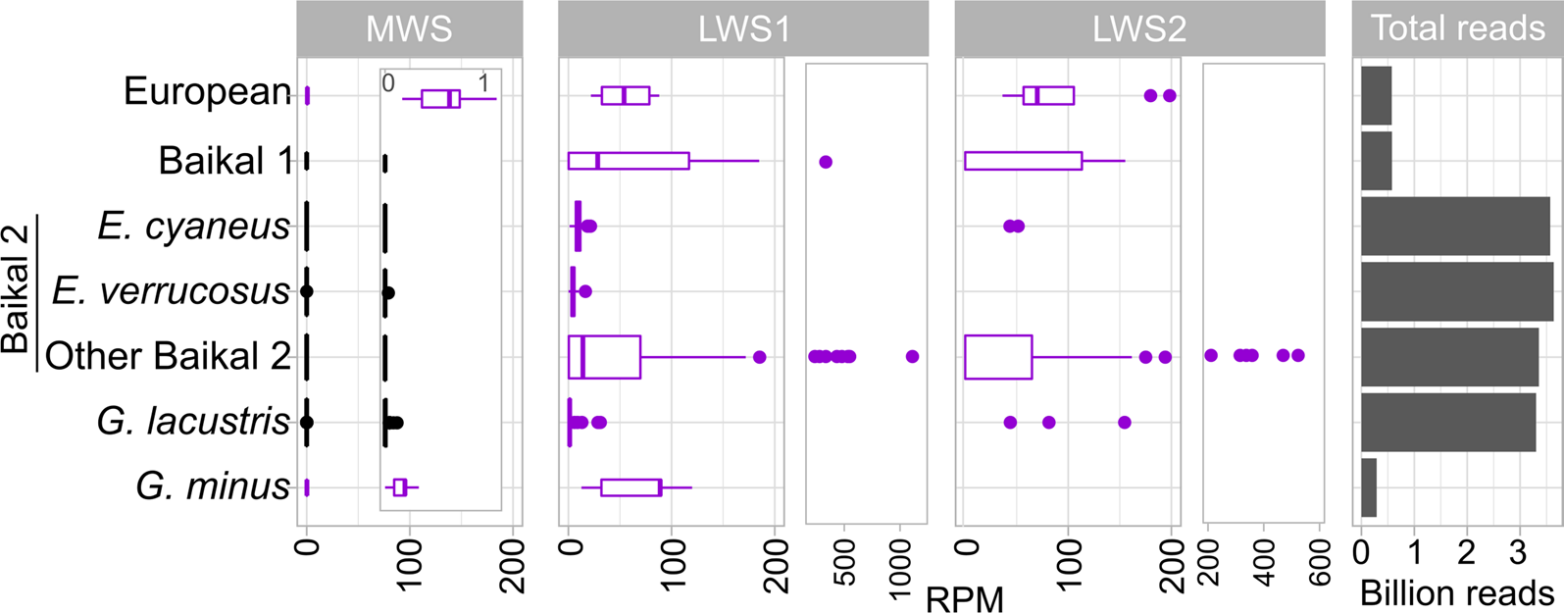
Expression levels of LWS and MWS opsins in Gammaridae estimated by the alignment of raw sequencing reads of selected species to the nucleotide sequences of one MWS and two LWS opsins found in the transcriptome of *G. pulex*. European = European freshwater species (*G. pulex*, *G. fossarum*, and *G. wautieri*). Boxplots are violet-coloured if the median was positive and black-coloured if it was equal to zero. RPM, reads per million

According to the phylogenetic analysis, *G. pulex*, *G. fossarum*, and *G. wautieri* were the closest species to Baikal amphipods possessing MWS opsins. Thus, we used the opsin sequences from *G. pulex* as a reference to align raw reads from Baikal amphipods and other Gammaridae and search for opsin reads. The results (Fig. 3, Additional file 4: Table S2) generally confirmed the findings made with opsin contig search (Additional file 1: Table S1) but provided deeper insights into the diversity of opsins. First, in European freshwater gammarid species, in which the expression of MWS opsins was indeed detected, its level was approximately three orders of magnitude lower than the expression level of LWS opsins (Fig. 3). Analysis of the contig coverage values provided by rnaSPAdes in the fasta headers (Additional file 5: Text S2) result in the same conclusion: the expression of LWS and MWS opsin transcripts differs approximately 100-fold. Second, in all of the comparatively low-coverage sequencing data for over 60 species of Baikal amphipods, totalling over 0.5 billion reads for the Baikal 1 group and over 3 billion reads for the Baikal 2 group, no MWS reads were found [47]. In the case of deep resequencing of Baikal species *E. cyaneus* (over 3 billion reads) [48], no reads aligned to MWS opsins, and in the case of *E. verrucosus* reads from the same study (also over 3 billion reads), only two reads (one read pair) aligned to the MWS opsin. These two reads may even have originated from *G. lacustris* material, as *G. lacustris* samples were present in the same study [48] and the same sequencing run.

These data were additionally confirmed *in vitro*. We amplified fragments of MWS and LWS opsins from cDNA samples of several species from the both the first Baikal clade (*Gm. fasciatus*, *Micr. wahlii platycercus*, and *Macr. branickii*) and the second Baikal clade (*E. cyaneus* and *Omm. albinus* with primers designed to anneal to conservative sequences in gammarid opsins (Additional file 6: Table S3). The results confirmed the presence of LWS opsins and absence of MWS opsins in Baikal amphipods (Additional file 2: Figs. S3 and S4), while the sample of *G. pulex* used as a positive control indeed showed MWS opsin expression.

Interestingly, in some other *Gammarus* lineages, we observed reads aligning to the *G. pulex* MWS opsin. Some reads aligning to the MWS opsin sequence were found in approximately one-quarter of the *G. lacustris* samples, and in all samples of *G. minus* (Additional file 2: Fig. S3). A possible explanation for this difference could be that in *G. lacustris* and *G. minus* the expression of MWS opsins is mostly transcriptionally repressed but still possible, while in Baikal lineages it is fully repressed or the genes are missing. To check the presence of MWS opsin sequences in *G. lacustris*, we extracted DNA from samples of this species and tried to amplify LWS and MWS opsins. Indeed, we detected a reproducible signal from one of the primer pairs in *G. lacustris* samples (Additional file 2: Fig. S3). These data support the idea that in *G. lacustris*, at least part of the MWS opsin gene was retained.

Taken together, the analyses of transcriptome assemblies and read alignment confirm at least two independent losses of MWS opsin expression in Baikal amphipods.

### Extraocular opsin expression is present in amphipods

Some of the available data represented transcriptomes of parts of animals that could not include eyes, such as the muscle tissues of *Eog. possjeticus* [71] and the pereon and pleon of *Hir. gigas* [72]. In the case of *Eog. possjeticus*, an LWS opsin was found in the assembly (Additional file 1: Table S1). In the case of *Hir. gigas*, no full-length opsin transcripts we found in the assembly, but the alignment of raw reads from pereon and pleon revealed the presence of reads matching the opsin recovered from an assembly based on whole-body material (Additional file 2: Fig. S4).

These results hint at the presence of extraocular opsin expression in amphipods. To test if it is present in Baikal amphipods, we extracted RNA from heads and the rest of the body of *E. cyaneus* and *O. albinus* individuals and checked the expression of opsins with RT-PCR. Indeed, in both the head and the rest of the body we found LWS opsin expression (Additional file 2: Fig. S4; Table 1).

**Table 1:** Evidence for extraocular expression of opsins in amphipods

Species	Tissue / body part	Method	Data source	LWS opsin detected?
*Eog. possjeticus*	Muscle	PIA3	NCBI TSA [71]	Yes
*Hir. gigas*	Pereon + pleon	Read alignment	NCBI SRA [72]	Yes
*E. cyaneus*	Pereon + pleon	RT-PCR	This work	Yes
*O. albinus*	Pereon + pleon	RT-PCR	This work	Yes

### Behavioural experiments do not reveal parts of the human visible light spectrum to which Baikal amphipods are insensitive

The presence of only LWS opsins raises the question of which regions of the light spectrum the Baikal amphipods can perceive and use for guiding their locomotion. The crustacean LWS pigments studied with microspectrophotometry have absorption maxima at 496–533 nm; their absorption spectra are quite wide and can easily cover the region between 400 and 600 nm [31]. To provide at least an indirect answer to this question, we exploited two previously reported behavioural reactions of Baikal fauna to light.

First, many species of Baikal amphipods are known to perform diel vertical migration, being a part of the night migratory complex [45, 46]. Practically, this means that at night amphipods are attracted by light. Moreover, there are some differences in how much the pelagic amphipod *M. branickii* is attracted to different light sources with wide and only partially overlapping spectra [73]. To check how the amphipods would react to narrow parts of the visible light spectrum, we performed field studies submerging light traps in Baikal. The animals were attracted by all the light sources (blue, green, yellow, and red; see Additional file 2: Fig. S5 for spectral characteristics) that we used. The traps with no light source contained less than 0.4% of the total animal count in light traps, while the traps with blue, green, yellow, and red light-emitting diodes (LEDs) contained 19.1%, 25.2%, 22.9%, and 32.5% animals, respectively (Additional file 7: Table S4). The overwhelming majority of individuals were small (< 1 mm-long and thus hard to identify) juveniles of *Micruropus* sp. and similar genera; we also encountered adult-sized *Micr. wahlii*, juvenile and adult-sized *Gm. fasciatus*, juvenile *Eulimnogammarus* sp., and *M. branickii*. Generally, these data show that Baikal amphipods are attracted to different wavelengths and therefore are able to perceive them.

**Fig. 4:**
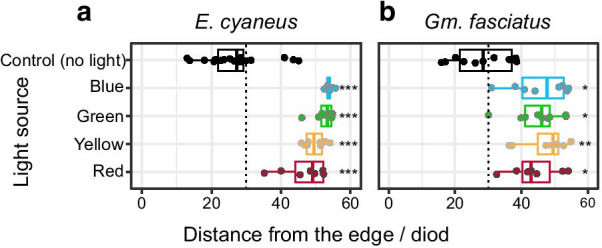
The results of laboratory experiments in *E. cyaneus* (**a**) and *Gm. fasciatus* (**b**) show avoidance reactions to different parts of the visible light spectrum. The centres / half widths at half maxima of the spectra of the blue, green, yellow and red LEDs are 457/11, 519/18, 593/8, and 626/8, respectively. Each dot represents the median of one experiment (20 animals). *Stands for p < 0.05 and **stands for p < 0.01 (Mann–Whitney test vs. the control level with Holm’s correction for multiple comparisons)

Second, gammarid amphipods generally show negative phototaxis in laboratory settings [74–76]. Light avoidance behaviour has also been used to assess the effect of chemicals in Baikal amphipods; these studies used white light [77]. For this test, we chose two species, *Gm. fasciatus* and *E. cyaneus* (first and second Baikal clades, respectively) with small body size and high locomotor activity. In both cases, we observed clear avoidance reactions to all presented light sources (Fig. 4). These data suggest that at least some shallow-living Baikal amphipods with multiple LWS opsins are able to perceive light both shorter than approximately 515 nm and longer than approximately 590 nm. It would be interesting to learn how deeper-living species with only a single LWS opsin transcript would respond under such experimental conditions.

### The diversity of LWS opsins

**Fig. 5:**
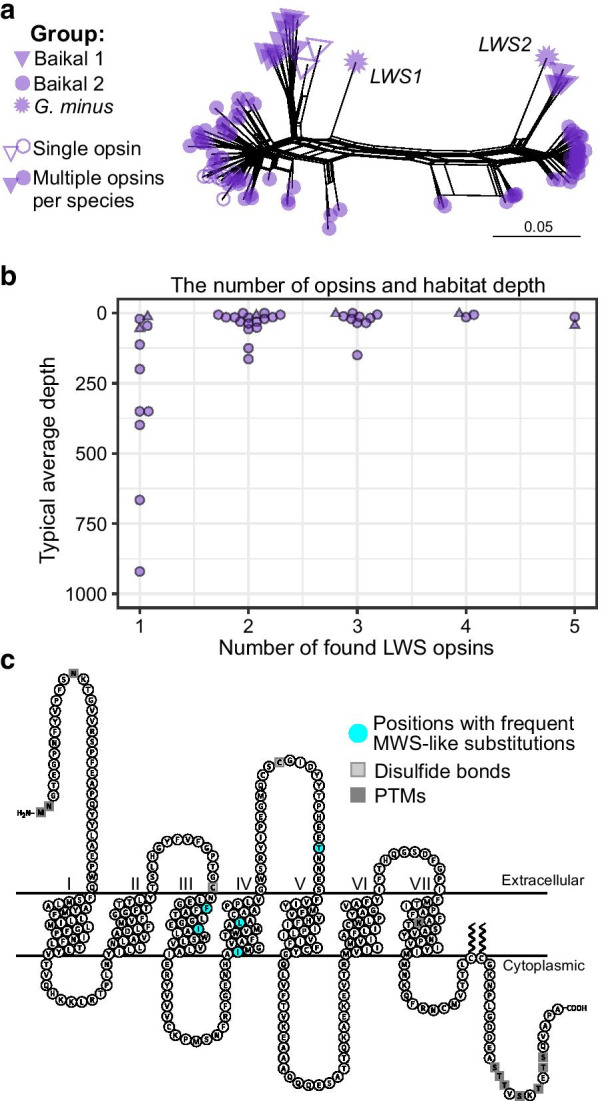
The diversity of LWS opsins in Baikal amphipods. **a** Only the species with at least one opsin detected were analyzed. The two opsins of *G. minus* are shown for comparison and classification. **b** The relationship between the number of opsins and habitat depth. The relationship is statistically significant (adjusted $\hbox {R}^{2}=0.17$; p = 0.003 in a linear model). For more information, see  Additional file 8: Table S5. **c** Frequent MWS-like substitutions in LWS opsins of Baikal amphipods superimposed on the secondary structure of bovine rhodopsin visualized with Protter [78]. For more information, see Additional file 9: Table S6.

As the majority of Baikal gammarids and all explored non-Baikal gammarids possess multiple LWS opsins (Additional file 1: Table S1), we hypothesize that several LWS opsins are the ancestral state for Gammaridae. To explore the diversity of LWS opsins, we built a phylogenetic network of nucleotide sequences. The LWS opsin sequences formed two clades, which were especially well-defined in Gammaridae (Additional file 2: Fig. S6; Fig. 5a), indeed suggesting that the last common ancestor of Gammaridae possessed two opsin genes.

A possible factor influencing the number of opsins could be the amount of light passing to the habitat depth of a particular species. Indeed, we found a significant negative correlation between the number of distinct opsin transcripts in the transcriptomes of Baikal amphipods and the typical average habitat depth of the corresponding species (Fig. 5b). Moreover, multiple opsins were only registered in species typically found above 200 meters, while most depths are occupied by species with one (or no) opsins.

Then, two processes should have taken place. First, some species, especially deep-water ones, lost one of the LWS opsins. Intriguingly, in both groups, the remaining single opsin per species belonged to the LWS1 clade. It might be a coincidence or mean that LWS2 is located in a less favourable genomic environment and is thus more likely to be lost. Second, even more species had more than two opsins, suggesting gene duplication and possible neofunctionalization, including spectral tuning that would shift the peak absorbance towards shorter wavelengths.

To test this hypothesis, we looked for MWS-like amino acid substitutions in LWS sequences of Baikal amphipods. MWS-like substitutions were defined as amino acids found in the same positions in LWS sequences of Baikal amphipods and in MWS sequences of European *Gammarus* sp., but not in LWS sequences of the European *Gammarus* sp. (Additional file 9: Table S6). We found 32 such substitutions, five of which were encountered more than 10 times (Fig. 5c). The most common MWS-like substitutions were encountered 48, 30, and 19 times, respectively. Interestingly, these three substitutions co-occurred in 11 sequences out of 102 total Baikal LWS sequences, which is much higher than 2.6% sequences expected with three independent events. The second most common substitution occurred in position 115 (bovine numbering), which has been implicated in spectral tuning in butterfly LWS opsins [79]. In addition, some of the sequences contained MWS-like substitutions in positions 90, 109, and 123, which have also been implicated in spectral tuning in invertebrates [79, 80]. Even though additional evidence is needed before drawing conclusions about the spectral sensitivity of LWS opsins in Baikal amphipods, these data show that they have the potential for spectral tuning.

## Discussion

In this work, we explored the diversity of opsins in amphipods and found that several amphipod lineages, including two independent invasions into Lake Baikal, express only LWS opsins. In contrast to Baikal amphipods, gammarid species closely related to them possess LWS and MWS opsins, even though the expression level of the latter is about two orders of magnitude lower. This difference in expression levels may mean that we witness the process of MWS opsin extinction in European gammarid species or that MWS opsins play a specific role in colour vision while LWS opsins have more diverse functions. Interestingly, we found evidence for LWS opsin expression in multiple body parts. This result might reflect opsin expression throughout the nervous system, which is indeed common in crustaceans [81, 82], or even suggest additional functions in this protein class, as, for example, an extraocularly expressed LWS opsin was already found in ovaries and probably regulated ovarian maturation in a decapod prawn *Macrobrachium nipponense* [83]. The nature of this phenomenon clearly requires further studies.

As LWS opsins of all gammarids form two clear clusters, all MWS form one cluster, and no cases with multiple MWS opsins per species have been found, it is logical to suggest that the last common ancestor of gammarids possessed one MWS opsin gene and at least two LWS opsin genes (Additional file 2: Fig. S6; Fig. 5a). The ancestral state reconstruction analysis also supports these hypotheses (Additional file 2: Figs. S2C,D). Both lineages that formed after the invasion into Baikal lost MWS opsin expression, deep-water species lost at least one of LWS opsins, while some other species underwent opsin multiplication, leading to three to five distinct transcripts per species. Markedly, this multiplication is especially prominent in the first (older) lineage, possibly due to their longer evolutionary history in Baikal or the fact that all species with sequenced transcriptomes inhabit relatively shallow depths.

The repeating two-step evolutionary scenario in both Baikal lineages (the loss of MWS opsins and subsequent restoration of opsin diversity via LWS opsin duplication) suggests common triggers. There are hypotheses that at some periods of Lake Baikal geologic history the shallow euphotic depths were uninhabitable, and the fauna survived only in deep-water refugia [47]. However, amphipods inhabiting deep-water environment express only one LWS opsin as we observe with certain Baikal species and marine *Hir. gigas*. Thus, our data suggest that at some points the ancestors of both Baikal amphipod lineages inhabited a relatively, but not completely dark environment. Such an environment could exist under ice [84].

Geological evidence suggests the longest cold episodes occurred at 2.82–2.48 and 1.75–1.45 million years ago [85]. During these periods, primary production in the lake decreased significantly, and around 2.67 million years ago glaciers extended into the lake [85].

Our results go in line with these data and allow us to hypothesize that the loss of MWS opsins occurred during such ice period(s) when colour vision was less important due to low amount of available light and its narrow spectral composition. Interestingly, the Baikal sculpins (Pisces: Cottidae), another endemic species flock inhabiting all depths in Baikal, show a depth-dependent opsin diversity but do not show any opsin gene losses common for the whole group [42]. According to the molecular data, the age of Baikal sculpins is estimated between 6.5 and 1.2 million years [86, 87], while the Baikal amphipods may be much older (10–30 million years) [40, 70, 88]. Thus, the latest long-term reduction in available light possibly occurred between the introgression of the ancestor of the younger group of Baikal amphipods and the introduction of the sculpin ancestor. Overall, these observations once again demonstrate the possibility of revealing climate history by following the evolutionary changes in protein families.

## Conclusions

In this work, we provide a comparative analysis of the diversity of opsins in approximately 90 species of amphipods (Crustacea: Amphipoda) and conclude that it is generally restricted to MWS and LWS opsins. The expression of LWS opsins was found outside the eye tissues, suggesting their expression throughout the nervous system and even possible multiple functions of these proteins. We evidenced (i) parallel loss of MWS opsin expression in multiple species (including two independently evolved lineages from the deep and ancient Lake Baikal) and (ii) LWS opsin amplification (up to five transcripts) in both Baikal lineages. The number of LWS opsins negatively correlated with habitat depth in Baikal amphipods. At the same time, some LWS opsins in Baikal amphipods contained MWS-like substitutions, suggesting that they might have undergone spectral tuning. This repeating two-step evolutionary scenario suggests common triggers, possibly the lack of light during the periods when Baikal was permanently covered with thick ice and its subsequent melting, and demonstrates the possibility of revealing climate history by following the evolutionary changes in protein families.

## Materials and methods

All code used for data analysis is available at the Harvard Dataverse (https://doi.org/10.7910/DVN/XG1BJC), Dryad (https://doi.org/10.5061/dryad.fj6q573r9) and GitHub (https://github.com/AlenaKizenko/pia3_amphipod_opsins).

### Data sources

All raw next-generation sequencing data used in this study were downloaded from public repositories. Raw sequencing reads and the corresponding transcriptome assemblies, whenever the latter were available, were downloaded from the sequence read archive (SRA) and transcriptome shotgun assembly (TSA) NCBI databases, respectively, with the fastq-dump command from NCBI SRA toolkit v2.9.2 (http://ncbi.github.io/sra-tools/). The accession numbers and corresponding references are provided in Additional file 1: Table S1.

### Sequence data analysis

The raw RNA-seq reads downloaded from the SRA database were used to recreate transcriptome assemblies. Data quality control was performed with FastQC (http://www.bioinformatics.babraham.ac.uk/projects/fastqc) v0.11.8 and summarized with MultiQC [89] v1.2. Read trimming was performed with trimmomatic [90] v0.36 in the pair-end mode. Transcriptome reassemblies were obtained with Trinity [91] v2.8.5 or rnaSPAdes [49] v3.13.1. Assembly completeness was estimated with BUSCO [92] v3.0.2. Reassembled data are available from the Harvard Dataverse (https://doi.org/10.7910/DVN/XG1BJC).

PIA3 is based on PIA [93] and PIA2 [15]. It uses python3 and standard python packages; specialized python packages Biopython [94] v1.77 and ETE3 [95] v3.1.1; TransDecoder [91] v5.5.0; standalone ncbi-blast+ [96] v2.10.1, diamond [97] v0.9.24; cd-hit [98, 99]; and Snakemake [100, 101]. The principle of PIA3 is illustrated in Additional file 2: Fig. S7. The useful features of PIA3 are the following.

- PIA3 should be easily installed on any UNIX-like platform. Prerequisites are general-purpose software packages Python3, conda, and Snakemake [100]. The required versions of all specialized packages are installed automatically at the first use.
- We tried our best to make the algorithm user-friendly by commenting the code and writing meaningful error messages.
- PIA3 uses cd-hit [98, 99] to report one sequence for a cluster of >95% identical sequences (clustering can be turned off or threshold can be altered by the user). This option allows easy calculation of the number of opsins from as many assemblies of the same species as needed within one run.
- It is possible to report only reasonably long sequences (those starting with the methionine codon and having length greater or equal than the mean length of the database). This behaviour can be turned off.
- PIA3 checks if the sequences contain the lysine residue in the position corresponding to $\hbox {K}^{296}$ in bovine rhodopsin, which is characteristic for opsins, and also assign classes according to the user-provided database. This behaviour can also be turned off.
- The database is an amino acid fasta file provided by the user (the database provided with the packaged version included most of the full-length opsins of crustaceans available at the time of writing, so the packaged version of PIA3 is tailored to specifically look for opsins in crustaceans).

Non-redundant sets of amino acid and nucleotide opsin sequences found with PIA3 are available in Additional file 3: Text S1 and Additional file 5: Text S2, respectively.

The species phylogenies were reconstructed with rnaSPAdes assemblies for one species per each genus (excluding the formal genus *Gammarus*) to avoid artefacts due to biased taxonomic sampling; only the assemblies of sufficient quality (over 50% complete BUSCOs) were used for the analysis. Protein sequences were predicted with TransDecoder v5.5.0 with the ---single_best_only option enabled. After removing redundancy with cd-hit v4.8.1-2019-0228 with a 95% identity threshold (-c .95), the protein sequences were clustered with proteinortho [102] v6. Then, either the amino acid sequences or the corresponding coding sequences were extracted for each orthologous group with custom code. Then, the sequences of each gene/protein family were aligned with mafft [103] v7.453; the alignments were quality trimmed with trimAl [104] with the -automated1 option and joined with seqkit. These concatenated trimmed alignments were used to build a maximum likelihood phylogeny with IQ-TREE [105] v1.6.10. The best-fit model was chosen automatically with ModelFinder ([106], and the topology was tested using 1000 Shimodaira-Hasegawa approximate likelihood ratio test (aLRT) bootstrap replicates and approximate Bayes (aBayes) tests [107, 108].

The amino acid-based opsin tree was created by aligning the sequences with mafft v7.453, trimming the alignment with trimAl and reconstructing a maximum likelihood phylogeny with IQ-TREE v1.6.10.

The ancestral state reconstruction analysis was performed with the phytools package [109] v0.7-70 for R [110] v.3.6.3 with the make.simmap function under the all rates different (ARD) model with 1000 simulations.

bowtie2 [111] v2.4.1 was used for the alignment of raw reads to the reference opsin sequences. Multiple sequence alignment was performed with mafft v7.453 for amino acid sequences or prank [112] v170427 in the codon mode for coding nucleotide sequences. UGENE [113, 114] v33 was used for manual inspection of multiple sequence alignments and short read alignments. kentUtils (https://github.com/ENCODE-DCC/kentUtils), pyfaidx [115] v0.5.8, samtools [116] v1.10, FASTX-toolkit (http://hannonlab.cshl.edu/fastx/toolkit/); SeqKit [117] and SnapGene Viewer (Insightful Science; available at snapgene.com) were also used for sequence file manipulation.

The figures were mostly created in the R programming environment [110] with the ggplot2 [118] v3.3.2; openxlsx (https://ycphs.github.io/openxlsx/); psycho [119]; phangorn [120]; ggtree [121, 122]; and other packages. iTOL [123] was also used to visualize phylogenetic trees, and SplitsTree [124] v4 was used to create phylogenetic networks from alignments.

### Field sampling and keeping the amphipods in the laboratory

Baikal amphipods (*E. cyaneus* and *Gm. fasciatus*) were sampled near the shoreline (depths of 0-1 m) in the Bolshie Koty village (south-west coast of Baikal; $51^{\circ }54'11.67''\hbox { N}$ $105^{\circ }4'7.61''\hbox {E}$) with kick-sampling (most littoral species), with fish-baited traps (*Omm. albinus*) or with light traps (*Micr. wahlii platycercus* and *Macr. branickii*). Representative of the Holarctic species *G. lacustris* were collected in the Irkutsk region (either Lake 14 in the vicinity of Bolshie Koty; $51^{\circ }55'14.39''\hbox {N}$, $105^{\circ }4'19.48''\hbox { E}$) or in a small water body in Irkutsk ($52^{\circ }16'05.1''\hbox {N }104^{\circ }16'56.6''\hbox {E}$). If needed, the amphipods were kept under laboratory conditions with constant aeration in the temperature matching the sampling temperature and weak ambient light. Fixation was performed in ethanol or by shock freezing in liquid nitrogen. Ethanol samples and frozen samples were stored at $-\,20^{\circ }\hbox {C}$ and $-\,80^{\circ }\hbox {C}$, respectively.

### Field and laboratory experiments

Field experiments (collection of amphipods with light traps) were performed in August 2019 after sunset (10.30 pm to 1 am). They were designed to test which parts of the light spectrum attract amphipods. It is well-established that many species of Baikal amphipods are attracted by light at night [45, 46], but these experiments used broad-spectrum white light.

For our experiments, we used waterproof LED strips with peaks in blue, green, yellow and red parts of the visible light spectrum. The spectra of the LED strips (Additional file 2: Fig. S5) were determined with a QE Pro fibre spectrometer (Ocean Optics, USA; not calibrated to a standard light source) with an attached F280SMA-A collimator (Thorlabs, USA). The strips were sequentially placed in the same position in a dark box in front of the collimator with an optical diffuser between them to reduce positioning inaccuracies.

To create a light trap, we placed an approximately 3.5 m-long LED strip into a 3-L plastic bottle (bottleneck diameter 5 cm) inside a light-tight cover (aluminium foil and black fabric). The bottle either held horizontally parallel to the shore at the depth of about 30 cm or fully submerged vertically at the depth of about 1 meter for 5–10 min and then raised. Each day, four traps with LED strips and one control bottle (without any LEDs or with a LED switched off) were submerged in random order; finally, the animals were collected in a nearby location with a hand net. Immediately after that, the samples were transported into the lab and sorted.

Laboratory experiments to test the reaction of amphipods to light were performed in a glass tank 60 × 10 × 10 cm half-filled with pebbles, on top of which we placed plastic food wrap filled with water. Twenty animals were placed there, mixed with a spoon and presented with a light source at a side of the tank. To exclude other environmental factors, both symmetrical configurations (LED strips on the left or right side) and no light stimulus (control) configuration were tested. After 1 min, we switched on the light and immediately took the photograph. The coordinates of each animal were determined manually with GIMP (www.gimp.org) v2.8.22 and quantified with ImageJ (Fiji) [125, 126] v1.52p.

### Nucleic acid extraction and PCR

The conclusions drawn from the bioinformatic analyses were checked with RNA/DNA extraction and subsequent PCR. Detailed information about the samples used for RNA or DNA extraction is available in Additional file 10 Table S7. RNA was extracted by homogenizing frozen amphipod tissues in Trizol (MRC, Germany) with 3/5-mm stainless steel beads (Qiagen, Germany) in a TissueLyser LT (Qiagen, Germany) and centrifuged. The supernatant was mixed with chloroform; phase separation was done in MaxTract high-density tubes (Qiagen, Germany). Total RNA was then purified with an RNeasy mini kit (Qiagen, Germany) according to the manufacturer’s recommendations, devoid of residual genomic DNA with a RapidOut DNA removal kit (Thermo, Lithuania), and subjected to cDNA synthesis with a Reverta reverse transcription kit (AmpliSens, Russia) with random primers. DNA extraction was performed from ethanol-conserved or frozen samples with DNA-Sorb-M kit (Amplisens, Russia) according to the manual, except that the pre-treatment included lasted 16 h at $+64^{\circ }\hbox {C}$. PCR amplification was performed with a 5× Screen Mix (Evrogen, Russia) and the following program: $95^{\circ }\hbox {C}$ for 5 min; 30 cycles of $95^{\circ }\hbox {C}$ for 30 s, $58^{\circ }\hbox {C}$ for 45 s, and $72^{\circ }\hbox {C}$ for 1 min; and $72^{\circ }\hbox {C}$ for 5 min. Primer sequences are listed in Additional file 6: Table S3, and binding sites are visualized in Additional file 2: Fig. S3. PCR product bands, as well as the quality and integrity of DNA and RNA, was visualized with TAE-agarose gel electrophoresis.

## Supplementary information

**Additional file 1: Table S1**. Transcriptome assemblies used in this work (including data sources, assembly completeness, and the number of expressed opsins).

**Additional file 2: Figure S1.** Quality of transcriptome assemblies according to BUSCO metrics (A-E) and the number of opsins found in each assembly (F). **Figure S2.** Amino acid-based phylogenetic trees of opsin sequences and amphipod species. (A) An amino acid-based maximum likelihood tree of all found opsin sequences and reanalysis of long branches with NCBI BLAST. (C,D) Ancestral state reconstruction analysis results for the number of MWS opsins (C) and LWS opsins (D). The pies represent the combined results of 1,000 runs of the simmap function (R package phytools) under the all rates different (ARD) model. The arrow points and the position of the last common ancestor of Gammaridae. **Figure S3.** Amplification of MWS and LWS opsins from genomic DNA (gDNA) and complementary DNA (cDNA) of several species. (A) Schematic of primer binding sites. (B,C) Opsin amplification from *G. pulex*, *G. lacustris*, *Micr. wahlii platycercus*, *M. branickii*, and *Gm. fasciatus* cDNA. (D,E) Coverage of the *G. pulex* MWS opsin with short RNA-seq reads of *G. minus* and *G. lacustris*. (F) Opsin amplification from genomic DNA of several species. Molecular weights of the DNA ladder bands are labelled in base pairs. The same ladder (100bp+, Evrogen) was used for (B), (C) and (E). **Figure S4.** Evidence for extraocular expression of opsins in amphipods. (A) Opsin amplification from cDNA of several species. Molecular weights of the DNA ladder bands are labelled in base pairs. (B) Expression of the H. gigas LWS opsin in the sample from pereon and pleon. **Figure S5.** Emission spectra of the LED light sources. The ticks and labels on the horizontal axis correspond to the centers of each spectrum and approximate borders of the human-visible light spectrum. The centres / half widths at half maxima of the spectra of the blue, green, yellow and red LEDs are 457/11, 519/18, 593/8, and 626/8, respectively. **Figure S6.** Phylogenetic network of all found amphipod opsins based on nucleotide sequences. **Figure S7.** The principle of PIA3.

**Additional file 3: Text S1**. Amino acid sequences of all discovered putative opsins.

**Additional file 4: Table S2**. Statistics of the alignment of short reads to the *G. pulex* opsins.

**Additional file 5: Text S2**. Nucleotide sequences of all discovered putative opsins.

**Additional file 6: Table S3**. Primers used in this work.

**Additional file 7: Table S4**. Species compositions of amphipods attracted to light traps deployed in the littoral of Lake Baikal.

**Additional file 8: Table S5**. The relationship between the number of discovered opsins, habitat depth, and assembly quality.

**Additional file 9: Table S6**. MWS-like substitutions in the LWS opsins of Baikal amphipods.

**Additional file 10: Table S7**. Information on sampling places and acclimation procedures for the individuals used for RNA or DNA extraction in this study.

## Data Availability

The data sets supporting the results of this article and code used to produce them are available in the Dryad repository [https://doi.org/10.5061/dryad.fj6q573r9] [127], in Harvard Dataverse [https://doi.org/10.7910/DVN/XG1BJC] [128] and GitHub [https://github.com/AlenaKizenko/pia3_amphipod_opsins].

## Abbreviations

LED: Light-emitting diode
LWS: Long wavelength-sensitive
MWS: Middle wavelength-sensitive
SWS: Short wavelength-sensitive
UVS: Ultraviolet-sensitive
