# Supplementary material for: The diversity of opsins in Lake Baikal amphipods (Amphipoda: Gammaridae)
Source: BMC Ecol Evol. 2021 May 10;21:81. doi: 10.1186/s12862-021-01806-9 (PMC8108468; doi:10.1186/s12862-021-01806-9)
Supplement: Supplementary file 2 — Additional file 2: Figure S1. Quality of transcriptome assemblies according to BUSCO metrics (A-E) and the number of opsins found in each assembly (F). Figure S2. Amino acid-based phylogenetic trees of opsin sequences and amphipod species. (A) An amino acid-based maximum likelihood tree of all found opsin sequences and reanalysis of long branches with NCBI BLAST. (C,D) Ancestral state reconstruction analysis results for the number of MWS opsins (C) and LWS opsins (D). The pies represent the combined results of 1,000 runs of the simmap function (R package phytools) under the all rates different (ARD) model. The arrow points and the position of the last common ancestor of Gammaridae. Figure S3. Amplification of MWS and LWS opsins from genomic DNA (gDNA) and complementary DNA (cDNA) of several species. (A) Schematic of primer binding sites. (B,C) Opsin amplification from G. pulex, G. lacustris, Micr. wahlii platycercus, M. branickii, and Gm. fasciatus cDNA. (D,E) Coverage of the G. pulex MWS opsin with short RNA-seq reads of G. minus and G. lacustris. (F) Opsin amplification from genomic DNA of several species. Molecular weights of the DNA ladder bands are labelled in base pairs. The same ladder (100bp+, Evrogen) was used for (B), (C) and (E). Figure S4. Evidence for extraocular expression of opsins in amphipods. (A) Opsin amplification from cDNA of several species. Molecular weights of the DNA ladder bands are labelled in base pairs. (B) Expression of the H. gigas LWS opsin in the sample from pereon and pleon. Figure S5. Emission spectra of the LED light sources. The ticks and labels on the horizontal axis correspond to the centers of each spectrum and approximate borders of the human-visible light spectrum. The centres / half widths at half maxima of the spectra of the blue, green, yellow and red LEDs are 457/11, 519/18, 593/8, and 626/8, respectively. Figure S6. Phylogenetic network of all found amphipod opsins based on nucleotide sequences. Figure S7. The principle [file 12862_2021_1806_MOESM2_ESM.pdf]

Supplemental Figures

Drozdova *et al.*

April 16, 2021

1 Diversity of opsin transcripts and their phylogenetic distribution

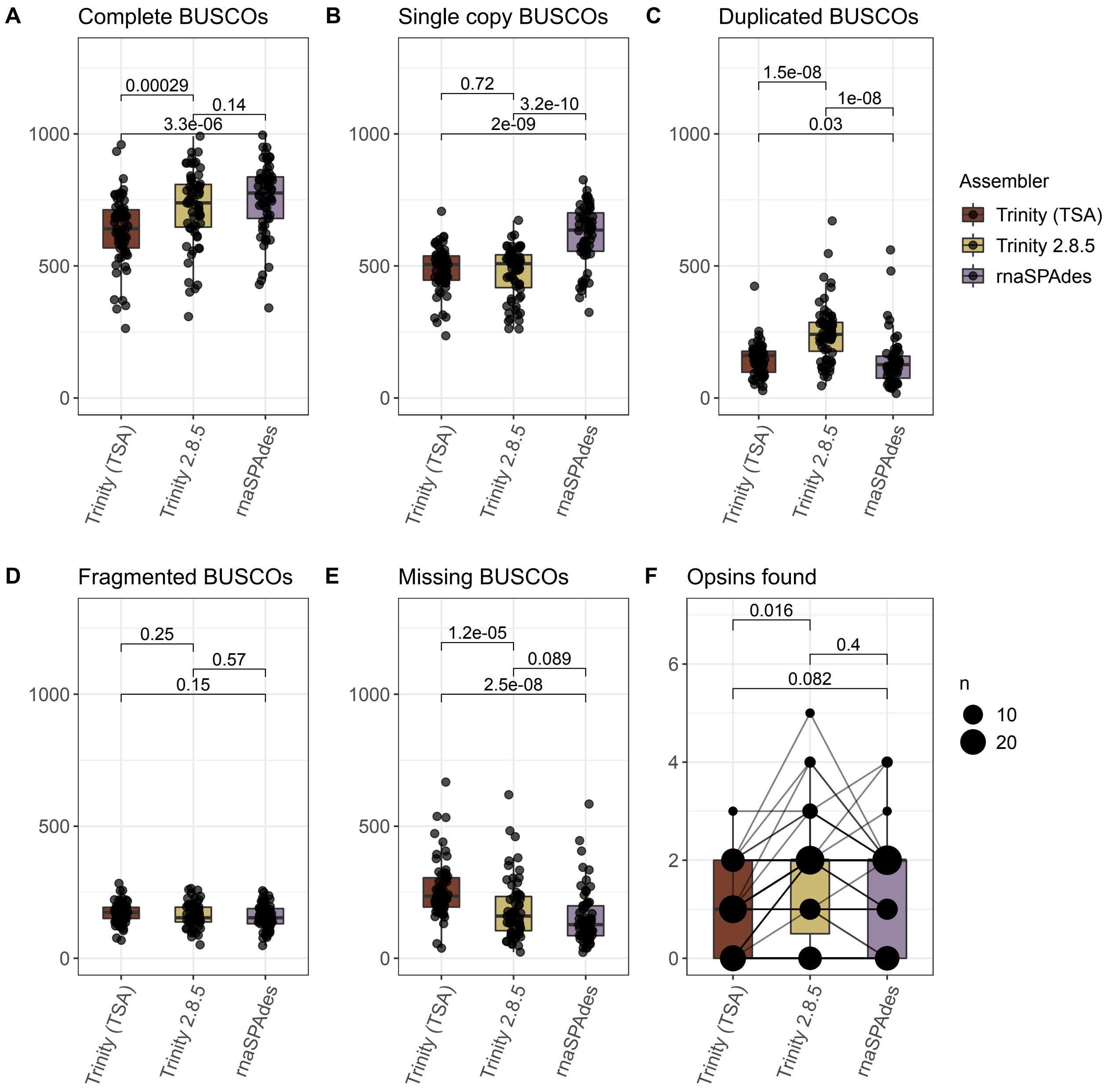

Figure S1: Quality of transcriptome assemblies according to BUSCO metrics (A-E) and the number of opsins found in each assembly (F)
.

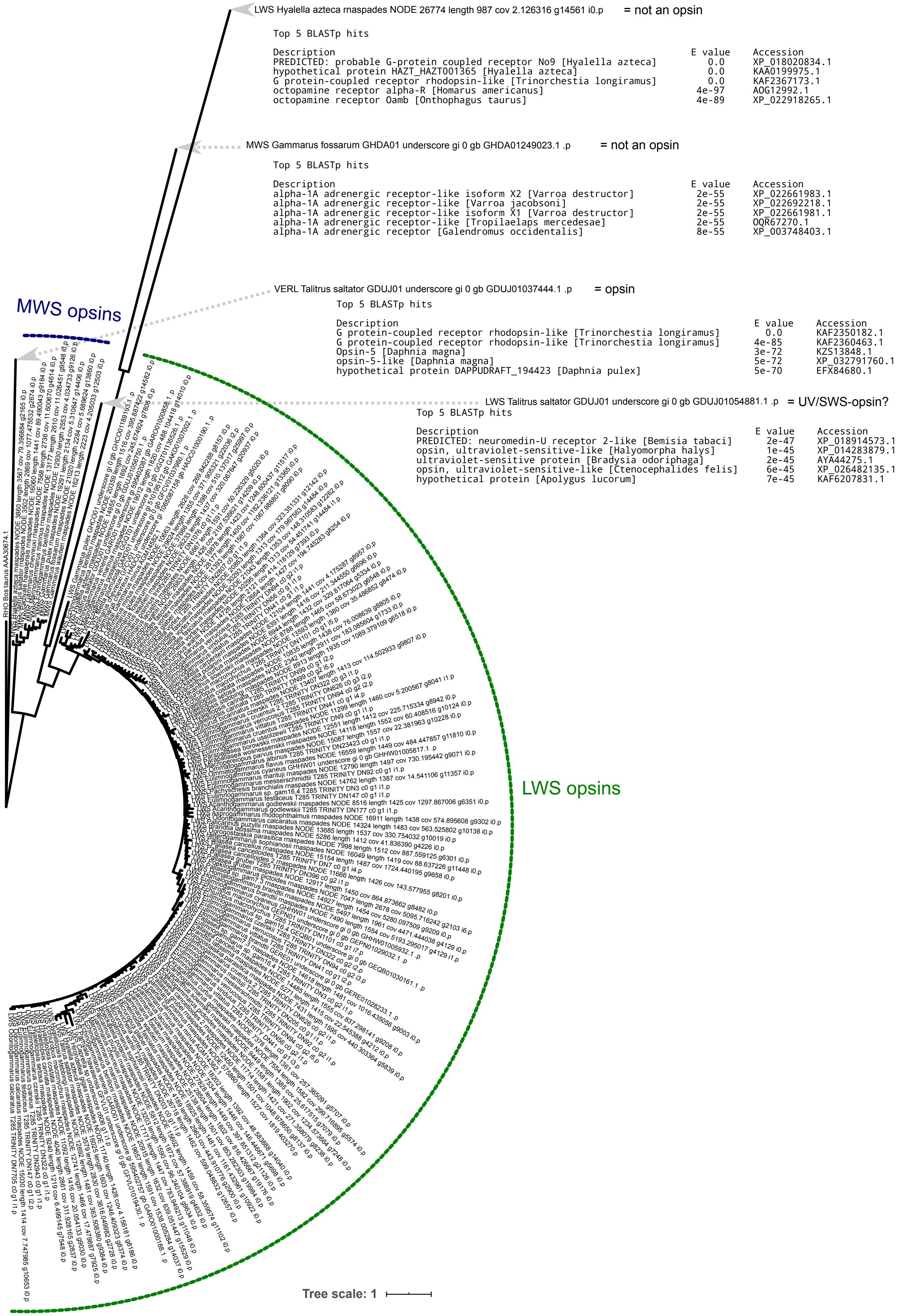

Figure S2: Amino acid-based phylogenetic trees of opsin sequences and amphipod species. (A) An amino acid-based maximum likelihood tree of all found opsin sequences and reanalysis of long branches with NCBI BLAST.

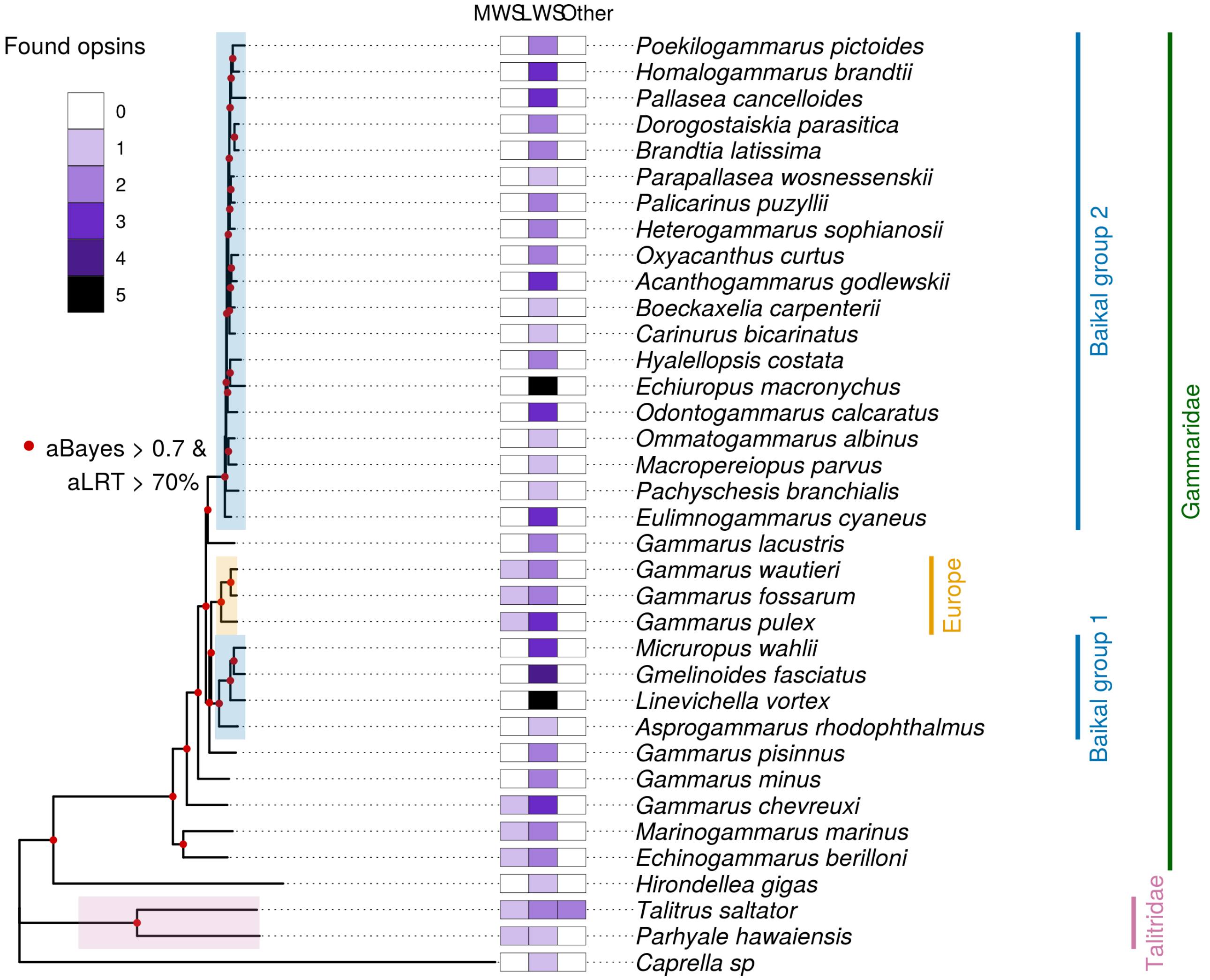

Figure S2: (B) Amino acid-based species tree based on one-copy orthologous proteins.

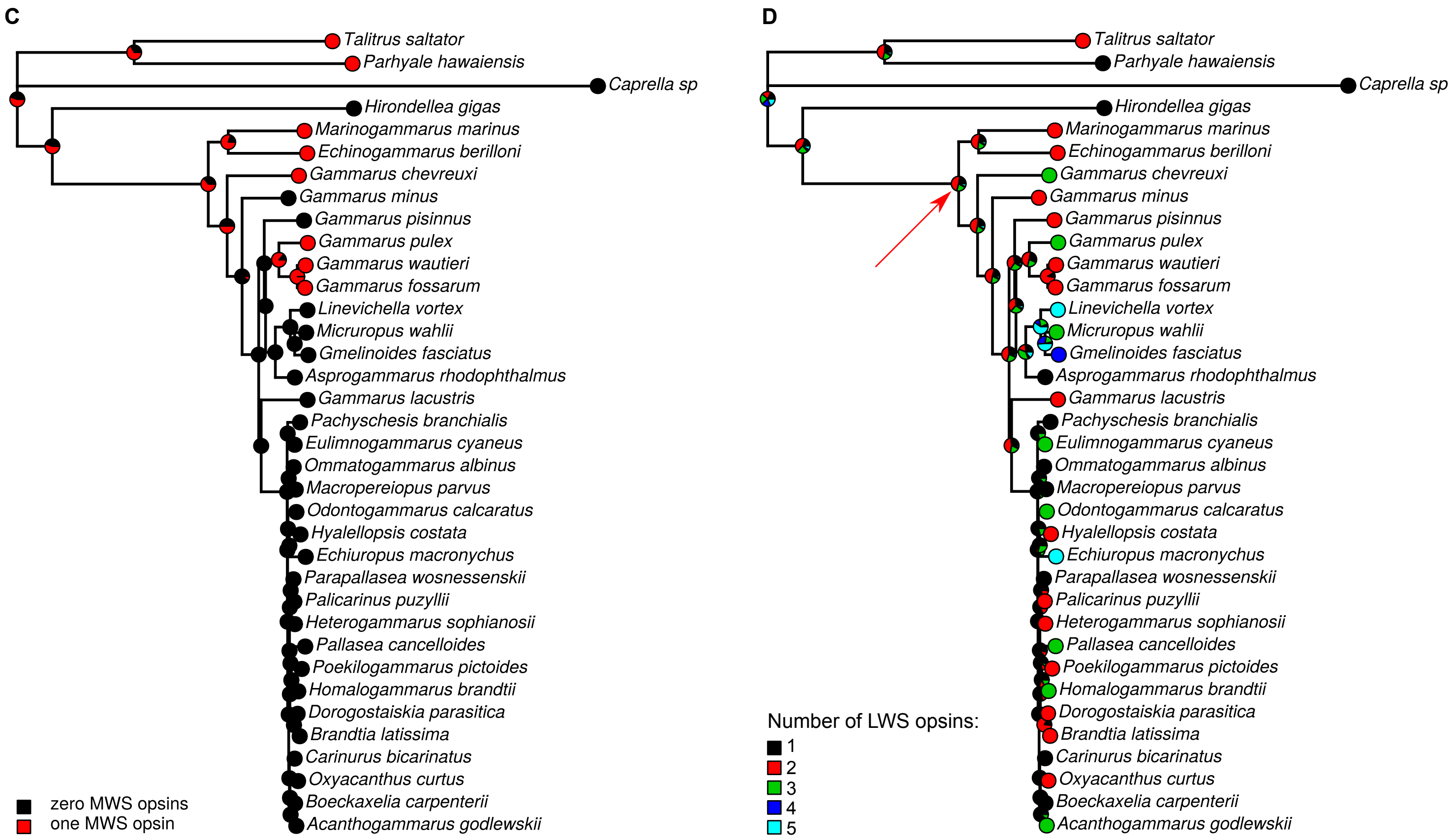

Figure S2: (C,D) Ancestral state reconstruction analysis results for the number of MWS opsins (C) and LWS opsins (D). The pies represent the combined results of 1,000 runs of the simmap function (R package phytools) under the all rates different (ARD) model. The arrow points and the position of the last common ancestor of Gammaridae.

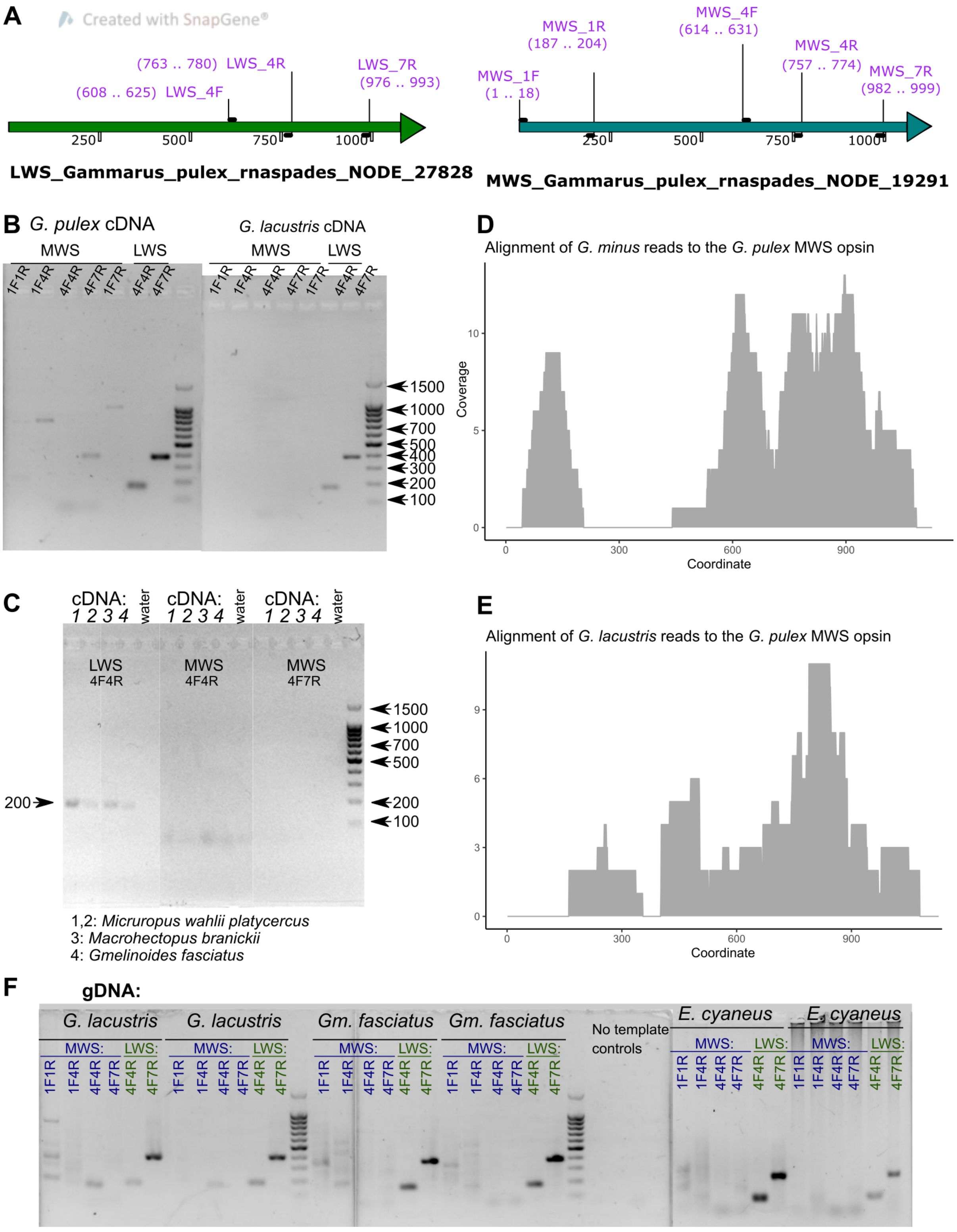

Figure S3: Amplification of MWS and LWS opsins from genomic DNA (gDNA) and complementary DNA (cDNA) of several species. (A) Schematic of primer binding sites. (B,C) Opsin amplification from *G. pulex*, *G. lacustris*, *Micr. wahlii platycercus*, *M. branickii*, and *Gm. fasciatus* cDNA. (D,E) Coverage of the *G. pulex* MWS opsin with short RNA-seq reads of *G. minus* and *G. lacustris*. (F) Opsin amplification from genomic DNA of several species. Molecular weights of the DNA ladder bands are labelled in base pairs. The same ladder (100bp+, Evrogen) was used for (B), (C) and (E).

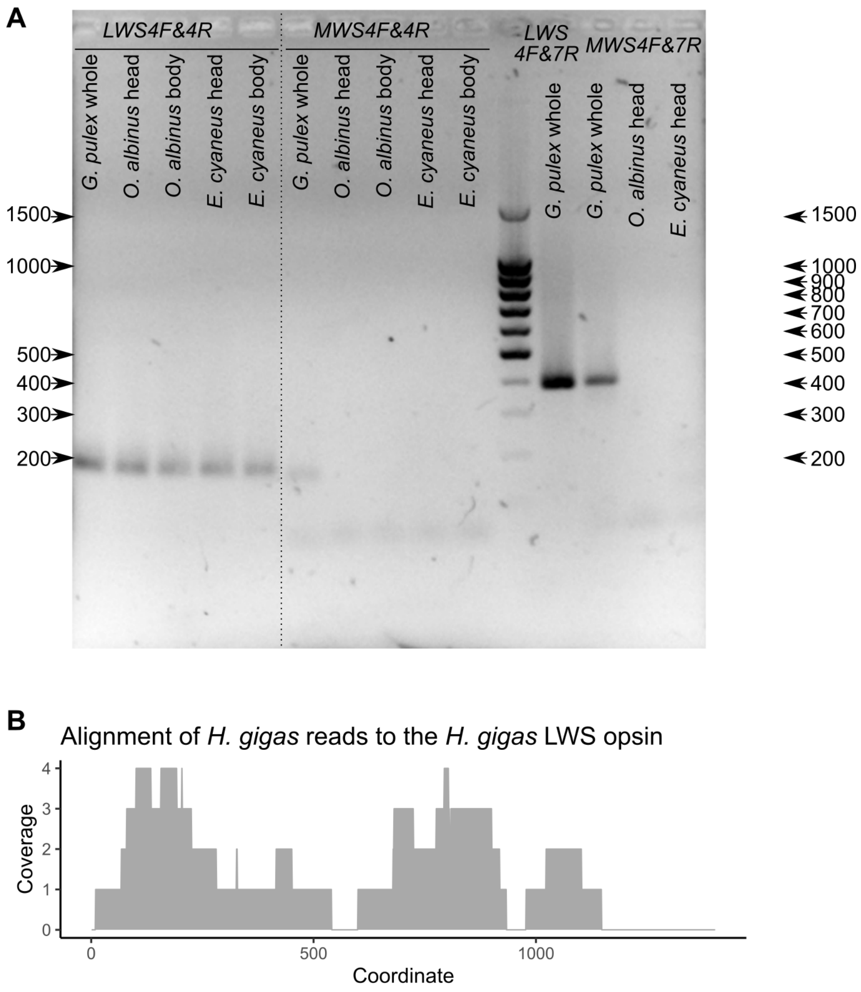

Figure S4: Evidence for extraocular expression of opsins in amphipods. (A) Opsin amplification from cDNA of several species. Molecular weights of the DNA ladder bands are labelled in base pairs. (B) Expression of the *H. gigas* LWS opsin in the sample from pereon and pleon.

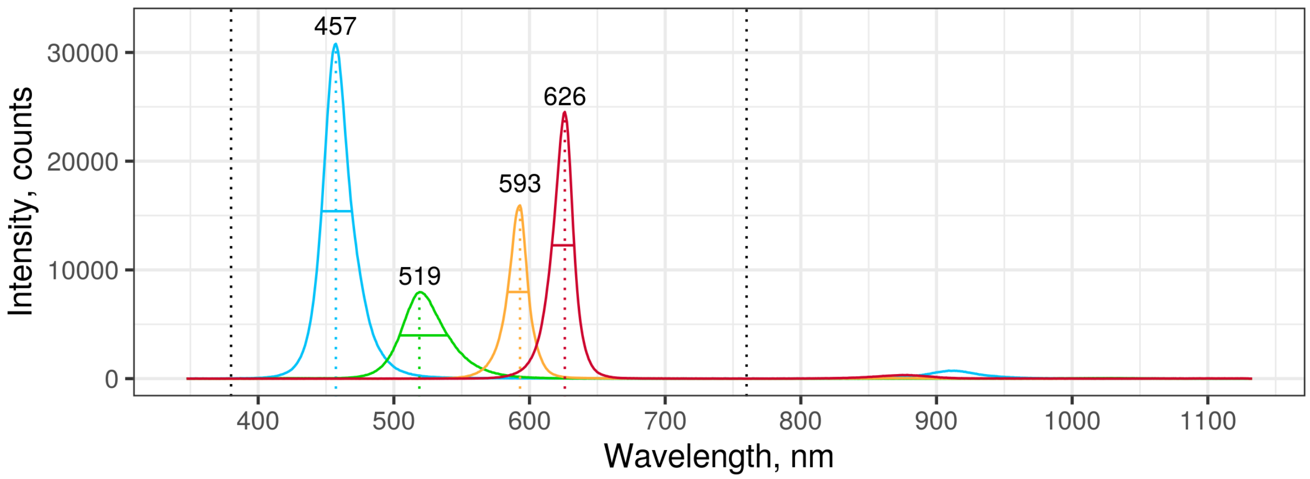

Figure S5: Emission spectra of the LED light sources. The ticks and labels on the horizontal axis correspond to the centers of each spectrum and approximate borders of the human-visible light spectrum. The centres / half widths at half maxima of the spectra of the blue, green, yellow and red LEDs are 457/11, 519/18, 593/8, and 626/8, respectively.

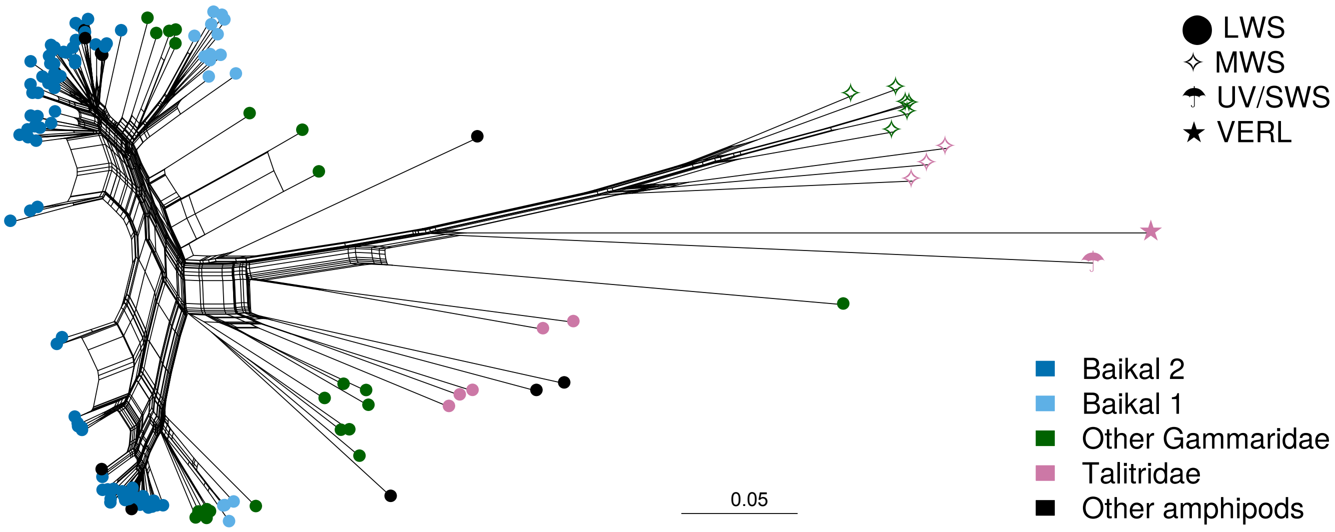

Figure S6: Phylogenetic network of all found amphipod opsins based on nucleotide sequences.

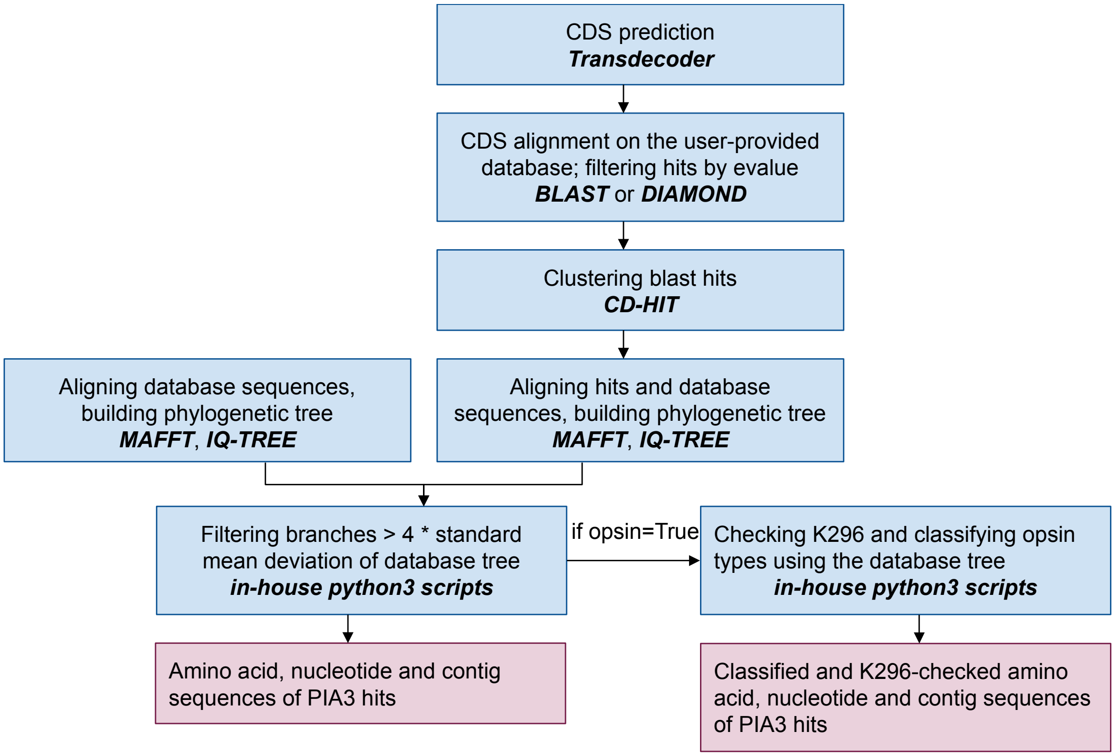

Figure S7: The principle of PIA3.
